# Supplementary material for: Glucose-1,6-Bisphosphate, a Key Metabolic Regulator, Is Synthesized by a Distinct Family of α-Phosphohexomutases Widely Distributed in Prokaryotes
Source: mBio. 2022 Jul 20;13(4):e01469-22. doi: 10.1128/mbio.01469-22 (PMC9426568; doi:10.1128/mbio.01469-22)
Supplement: TABLE S1 [file mbio.01469-22-s0002.docx]

| **Name** | **Typical length (aa)** | **Sugar-binding domain sequence motif** |
| --- | --- | --- |
| cd03085 (PGM1) | ~550 | **CGEESFG** |
| cd05799 (PGM2) | 550 – 650 | AF**EE**AIG |
| cd03089 (PMM/PGM) | ~450 | A**GEMS**G**H** |
| cd03087 (PGM_like1) | ~450 | G**GE**G**NG**G |
| cd05800 (PGM_like2) | ~450 | G**GEESGG** |
| cd05801 (PGM_like3) | ~550 | G**GEESAG** |
| cd05803 (PGM_like4) | ~450 | **GGEGNGG** |

**Table S1**: **PGM and “PGM like” subfamilies according to CDD:**

Given is the typical length and characteristic sugar-binding sequence motif of each PGM subfamily. Highly conserved amino acids are highlighted in bold
