# Supplementary material for: Glucose-1,6-Bisphosphate, a Key Metabolic Regulator, Is Synthesized by a Distinct Family of α-Phosphohexomutases Widely Distributed in Prokaryotes
Source: mBio. 2022 Jul 20;13(4):e01469-22. doi: 10.1128/mbio.01469-22 (PMC9426568; doi:10.1128/mbio.01469-22)
Supplement: TABLE S2 [file mbio.01469-22-s0003.docx]

|  | **cd03085** | **cd05799** | **cd03089** | **cd03087** | **cd05800** | **cd05801** | **cd05803** |
| --- | --- | --- | --- | --- | --- | --- | --- |
| *Mycobacterium tuberculosis* |  | + | + |  |  | + |  |
| *Escherichia coli* |  |  | + |  |  | + |  |
| *Helicobacter pylori* |  |  | + |  |  |  |  |
| *Bordetella pertussis* |  |  | + |  |  |  |  |
| *Neisseria gonorrhoeae* |  |  | + |  |  |  |  |
| *Eggerthella lenta* |  |  |  |  | + |  |  |
| *Bacteroides salyersisae* |  | + |  |  | + |  | + |
| *Synechocystis sp. PCC6803* |  |  |  |  | + | + |  |
| *Prochlorococcus marinus* | + |  |  |  | + |  |  |
| *Nostoc punctiforme* | + |  |  |  | + |  |  |
| *Deinococcus radiodurans* |  |  |  |  | + | + |  |
| *Geobacter metallireducens* |  |  |  |  | + |  |  |
| *Litorilinea aerophilia* |  |  |  |  | + | + |  |
| *Prosthecochloris vibrioformis* |  |  |  |  | + | + | + |
| *Granulicella mallensis* |  |  |  |  | + | + |  |
| *Ammonifex degensii* |  |  |  | + | + |  | + |
| *Vibrio cholerae* |  | + |  |  | + | + |  |
| *Caldicellulosiruptor owensis* |  |  |  |  | + |  |  |
| *Thermotoga caldifontis* |  |  |  |  | + |  |  |
| *Haloplanus aerogenes* |  |  |  | + | + |  |  |
| *Persephonella atlantica* |  |  |  |  | + |  |  |
| *Bacillus subtilis* |  | + |  |  |  |  |  |
| *Staphylococcus aureus* | + | + |  |  |  |  |  |

**Table S2: Distribution of PHM subfamilies considered to be PGM or “PGM_like” in different bacterial strains**

Blue color with (+) shows the presence of the respective αPHM subfamily in the listed strains
