# Supplementary figures and images for: Glucose-1,6-Bisphosphate, a Key Metabolic Regulator, Is Synthesized by a Distinct Family of α-Phosphohexomutases Widely Distributed in Prokaryotes
Source: mBio. 2022 Jul 20;13(4):e01469-22. doi: 10.1128/mbio.01469-22 (PMC9426568; doi:10.1128/mbio.01469-22)

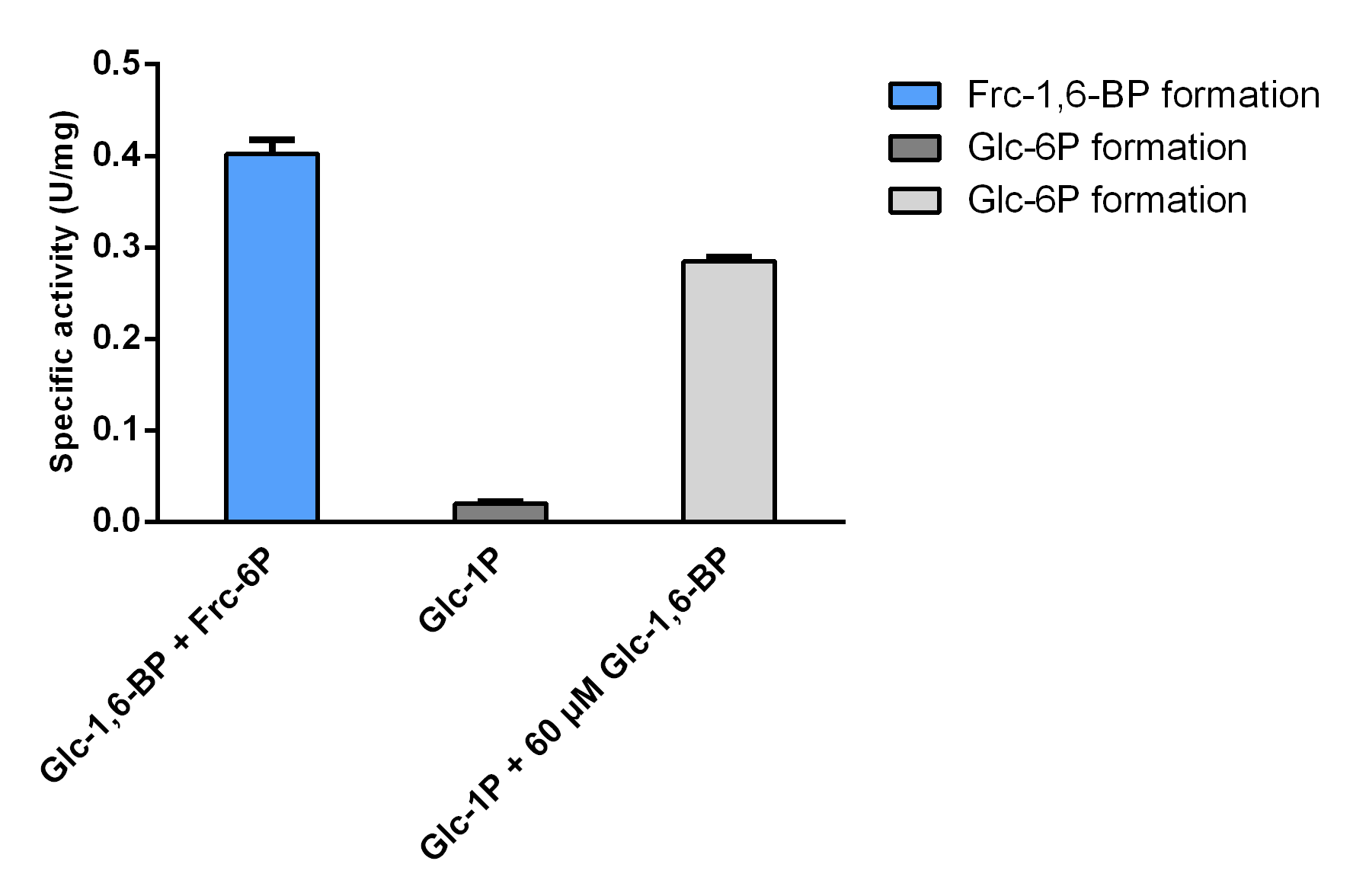

Supplement: FIG S1 [file mbio.01469-22-s0001.tif]
