# Supplementary material for: Glucose-1,6-Bisphosphate, a Key Metabolic Regulator, Is Synthesized by a Distinct Family of α-Phosphohexomutases Widely Distributed in Prokaryotes
Source: mBio. 2022 Jul 20;13(4):e01469-22. doi: 10.1128/mbio.01469-22 (PMC9426568; doi:10.1128/mbio.01469-22)
Supplement: TABLE S3 [file mbio.01469-22-s0004.docx]

| **Primer** | **Sequence (5’ - 3’)** |
| --- | --- |
| pASK-C(slr1334)_fw | TAGAAATAATTTTGTTTAACTTTAAGAAGGAGATATACAAATGGTTTACACTCCTGCTCC |
| pASK-C(slr1334)_rev | GGTCTTATTTTTCGAACTGCGGGTGGCTCCAGCTAGCCATGTGAGATGATTGTGCAGGCTTG |
| pASK-C(sll0726)_fw | TAGAAATAATTTTGTTTAACTTTAAGAAGGAGATATACAAATGACAAGCAGAATTAATCC |
| pASK-C(sll0726)_rev | GGTCTTATTTTTCGAACTGCGGGTGGCTCCAGCTAGCCATGCCCAAAGCCGAGGTAACAATG |
| pASK-IBA5_Cterm_fw | GGCTAGCTGGAGCCACCCGCAGTTCGAAAAATAAGACCATGGTCTCTGATATCTAACTAAG |
| pASK-IBA5_Cterm_rev | TTGTATATCTCCTTCTTAAAGTTAAAC |

**Table S3: List of the primers used in this study**
