# Supplementary material for: Glucose-1,6-Bisphosphate, a Key Metabolic Regulator, Is Synthesized by a Distinct Family of α-Phosphohexomutases Widely Distributed in Prokaryotes
Source: mBio. 2022 Jul 20;13(4):e01469-22. doi: 10.1128/mbio.01469-22 (PMC9426568; doi:10.1128/mbio.01469-22)
Supplement: TABLE S4 [file mbio.01469-22-s0005.docx]

| **Plasmid** | **Purpose** |
| --- | --- |
| pASK-C(sll0726) | Expression of Strep-tagged Sll0726 in *E.coli* |
| pASK-C(slr1334) | Expression of Strep-tagged Slr1334 in *E.coli* |
| pET15b(PGK) | Expression of His-tagged Phosphoglycerate-kinase in *E.coli* |

**Table S4. List of the plasmids used in this study**
