# Supplementary material for: Glucose-1,6-Bisphosphate, a Key Metabolic Regulator, Is Synthesized by a Distinct Family of α-Phosphohexomutases Widely Distributed in Prokaryotes
Source: mBio. 2022 Jul 20;13(4):e01469-22. doi: 10.1128/mbio.01469-22 (PMC9426568; doi:10.1128/mbio.01469-22)
Supplement: TABLE S5 [file mbio.01469-22-s0006.docx]

|  | | |  |
| --- | --- | --- | --- |
| **Strain** | **Genotype** | **Purpose** | **Manufacturer** |
| *E. coli* NEB10β | \| Δ(ara-leu) 7697 araD139 fhuAΔlacX74 galK16 galE15 e14-Φ80dlacZΔM15 recA1 relA1 endA1 nupGrpsL(StrR) rphspoT1 Δ(mrr-hsdRMS-mcrBC) \| \| --- \| | Molecular cloning | New England Biolabs  (Ipswich (Massachusetts), USA) |
| *E. coli Rosetta gami (DE3)* | Δ(ara-leu)7697 ΔlacX74 ΔphoA PvuII phoR araD139 ahpC galE galK rpsL (DE3) F'[lac^+^ lacI^q^ pro] gor522::Tn10 trxB pLysSRARE (Cam^R^, Str^R^, Tet^R^) | Protein overexpression | \|  \| Merck Millipore  ([Billerica](https://de.wikipedia.org/wiki/Billerica) ([Massachusetts](https://de.wikipedia.org/wiki/Massachusetts)),  USA) \| \| --- \| --- \| |

**Table S5. List of the bacterial strains used in this study**
